# How to Prevent Mammary Abscess

**Published:** 1880-09-20

**Authors:** S. S. Boyd


### HOW TO PREVENT MAMMARY ABSCESS.

BY S. S. BOYD, M.D.

In the September number of the American Practitioner, I find a very excellent paper, "A Simple Method of Preventing Mammary Abscess," copied from the Canada Medical and Surgical Journal.

For years past, when in my practice a mammary abscess is threatened, I have always applied a bandage to the breast with the most satisfactory results, never failing to prevent suppuration if resorted to in time. Even after the breast has suppurated at one point, if the bandage is properly applied, the extension of inflammation to other parts of the breast will be prevented; which was not the case when, under the old *regime*, I used all the poultices, plasters and liniments, which I and all the old women of the vicinity could suggest.

The bandage is applied as follows:

First, take a roller of sufficient length to pass around the waist of the patient just below the breasts. Then take a piece of strong muslin about eight inches square; cut a circular hole in the center of it, large enough to pass over the breast tightly; sew one edge of this to an edge of the band. Now raise the breast and gently draw the eight inch piece over it, so that the breast will protrude through the circular opening. Fasten the bandage as tightly around the waist as can be comfortably borne. From each upper corner of the square piece fasten a band and pass them over the shoulder, and secure tightly to the bandage which passes around the waist.

We now have a foundation for the superstructure, which we build as follows:

Take a soft roller, one and a half inches wide, and lifting the breast pass it around the breast two or three times at the base as tightly as can be tolerated by the patient. Then cut strips from the same roller about eight inches long, and fasten one end of one of the strips to the inner edge of the cloth through which the breast protrudes; fasten the other end of the strip to the opposite side of the ring, passing it over the breast but avoiding the nipple. Apply other strips in like manner until the breast, except the nipple, is completely covered; apply the strips as tightly as can be borne by the patient. It will astonish the uninitiated how much pressure an inflamed breast wili bear when thus gradually applied.

The same plan is resorted to when not called until suppuration is established, except that an opening in the bandaging is left at the point of exit of pus. When the bandages become loose, as they rapidly do, they should be tightened.

Not claiming that the above plan of treating mammary abscess is as neat as that given by Dr. Shepherd, I do claim that the bandaging more thoroughly fills what I take to be the indications in such cases, namely, to more effectually cut off the excessive supply of blood that feeds the inflammation, which once established often destroys the function of the breast, and brings almost intolerable sufferings to the patient. While I admit Dr. Shepherd's plan of treatment acts, as he says, by "exercising even pressure and giving support to it," yet I believe the facilities for renewing the pressure, as afforded by the foregoing suggestions, will be found essential to success in many cases.

Again, if we teach the student or young practitioner of medicine that by bandaging the breast we cure mammary abscess, he at once discovers that congestion, and even inflammation, can be overcome by pressure continuously applied; and he will not have to be taught that bandaging is the "sovereign thing on earth" for indolent ulcer, a sprain, or synovitis, and most other forms of inflammation where a bandage can be used or pressure applied. In short, we teach a principle which is adapted to a wide range of diseases, and not a mere fact which only applies to a single case.—*Amer. Practitioner.*

